# Whole-Genome Sequencing of *Bradyrhizobium diazoefficiens* 113-2 and Comparative Genomic Analysis Provide Molecular Insights Into Species Specificity and Host Specificity

**DOI:** 10.3389/fmicb.2020.576800

**Published:** 2020-11-16

**Authors:** Rong Li, Yong Feng, Haifeng Chen, Chanjuan Zhang, Yi Huang, Limiao Chen, Qingnan Hao, Dong Cao, Songli Yuan, Xinan Zhou

**Affiliations:** ^1^Key Laboratory of Biology and Genetic Improvement of Oil Crops, Ministry of Agriculture and Rural Affairs of PRC, Oil Crops Research Institute of Chinese Academy of Agriculture Sciences, Wuhan, China; ^2^State Key Laboratory of Agricultural Microbiology, Huazhong Agricultural University, Wuhan, China

**Keywords:** *Bradyrhizobium diazoefficiens* 113-2, whole-genome sequencing, comparative analysis, species specificity, host specificity

## Abstract

In the present study, we sequenced the complete genome of *Bradyrhizobium diazoefficiens* 113-2. The genomic characteristics of six selected rhizobial strains (two fast-growing rhizobia, two medium-slow-growing rhizobia and two slow-growing rhizobia) with four different legume hosts were analyzed by comparative genomic analysis. Genomes of *B. diazoefficiens* 113-2 and *B. diazoefficiens* USDA110 were found to share a large synteny blocks and a high ANI value, supporting 113-2 as a strain of *B. diazoefficiens*. 5,455 singletons and 11,656 clusters were identified among the six rhizobia genomes, and most of the pair-wise comparisons clusters were shared by the two genomes of strains in the same genus. Similar genus-specific gene numbers in the assigned COG functional terms were present in the two strains of the same genus, while the numbers were decreased with the increase of growth rate in most of the COG terms. KEGG pathway analysis of *B. diazoefficiens* 113-2 suggested that the rhizobial genes in ABC transporters and Two-Component system were mainly species-specific. Besides, the candidate genes related to secretion system and surface polysaccharides biosynthesis in the genomes of the six strains were explored and compared. 39 nodulation gene families, 12 *nif* gene families and 10 *fix* gene families in the genomes of these six strains were identified, and gene classes in most of gene families and the types and total gene numbers of gene families were substantially different among these six genomes. We also performed synteny analyses for above-mentioned *nod*, *nif*, and *fix* gene groupings, and selected *NodW*, *NolK*, *NoeJ*, *NifB*, *FixK*, and *FixJ* gene families to perform phylogeny analyses. Our results provided valuable molecular insights into species specificity and host specificity. The genetic information responsible for host specificity will play important roles in expanding the host range of rhizobia among legumes, which might provide new clues for the understanding of the genetic determinants of non-legume-rhizobium symbiosis.

## Introduction

Nitrogen-fixing symbioses between legumes and rhizobia provide the legume host with a large fraction of reduced atmospheric nitrogen in exchange for carbon source and shelter inside symbiosis-specific root nodules (Friesen, 2012). The efficiency of such cross-kingdom collaboration is mainly attributed to the symbiotic matching (symbiotic specificity), which is always associated with distinct nodulation phenotype (Jones et al., 2008; Hayashi et al., 2012; Yuan et al., 2016), leading to the existence of different legume-rhizobium associations. For example, *Mesorhizobium loti* MAFF303099 forms specific symbiosis with several host plants of *Lotus* (Estrella et al., 2009), *Mesorhizobium huakuii* 7653R can only form symbiosis with *Astragalus sinicus* (Wang et al., 2014), and *Sinorhizobium meliloti* can only nodulate *Medicago*, *Melilotus*, and *Trigonella* (Biondi et al., 2003; Radutoiu et al., 2007). The symbiotic specificity may be determined by a fine-tuned exchange of molecular signals between a host root and its inoculated rhizobial strains (Perret et al., 2000). These signals mainly include nodulation factors (NFs) (Lerouge et al., 1990; Schultze et al., 1992), surface polysaccharides (Skorupska et al., 2006; Jones et al., 2008) and secreted proteins/type III secretion system (T3SS) (Fauvart and Michiels, 2008; Okazaki et al., 2013; Nelson and Sadowsky, 2015). Lots of genes that affect the biological synthesis of these signaling molecules in the genomes of different strains have been explored by comparative genomics (Tian et al., 2012; Wang et al., 2014), and gene transfer between related taxa can alter the host range of symbionts (Temprano-Vera et al., 2018). Therefore, identifying the determinants responsible for host specificity plays important roles in expanding the host range of rhizobium.

According to the growth rate, rhizobia can be divided into fast-growing rhizobia (*Rhizobium*) (Keyser et al., 1982), slow-growing rhizobia (*Bradyrhizobium*) (Tampakaki et al., 2017) and medium-slow-growing rhizobia (*Mesorhizobium*) (Wang et al., 2014). Various genetic and environmental factors as well as the number of rRNA operons affect growth rates (Shrestha et al., 2007; Temprano-Vera et al., 2018; Cherni and Perret, 2019), yet carbon metabolism in cells with multiple carbon sources and high extent of carbon utilization in fast-growing rhizobia maybe tend to grow faster than others (Marsudi et al., 1999; Ansari and Rao, 2014). Compared with *Bradyrhizobium*, most of the *Rhizobium* have lower energy consumption as well as better environmental adaptability and nodulation competitiveness (Marsudi et al., 1999). *Mesorhizobium*, whose growth rate is intermediate between that of *Rhizobium* and *Bradyrhizobium*, is a genus of rhizobium with a narrow host range (Streit et al., 2004). An improved understanding of the genetic information differences among these rhizobia will provide molecular insights into understanding the characteristics of these three genera of rhizobia.

*Bradyrhizobium diazoefficiens* 113-2, a broad-host-range and highly efficient soybean rhizobium (isolated from soybean “monkey hair”), was collected from paddy fields in Hengyang area of Hunan Province, China in 1972 by Xuejiang Zhang, and it has been applied in sustainable agriculture in China, United States, and Canada. In our previous studies, *B. diazoefficiens* 113-2 had higher symbiotic matching abilities than *B. diazoefficiens* USDA110 and *Sinorhizobium fredii* USDA205 with soybean ‘Tianlong 1’ (Li et al., 2017b). The comparative analysis of symbiotic phenotypes of soybean ‘Tianlong 1’ with *B. diazoefficiens* 113-2 and *S. fredii* USDA205 (Li et al., 2017b) and the RNA-Seq analysis of differential gene expression responding to *B. diazoefficiens* 113-2 and *S. fredii* USDA205 in soybean roots (Yuan et al., 2016) have also been extensively studied. However, the genetic information of rhizobium responsible for the phenotypic differences among 113-2-soybean, *B. diazoefficiens* USDA110-soybean and USDA205-soybean associations, and different symbiotic matching abilities between 113-2-soybean and USDA205-soybean associations remains unclear, so comparative genomic analysis between *B. diazoefficiens* 113-2, *B. diazoefficiens* USDA110 and *S. fredii* USDA205 is an good ideal for discovering the genetic information of rhizobium related to the above-mentioned phenomenon.

In the present study, we investigated the entire genomic information of *B. diazoefficiens* 113-2 and provided useful insights into this strain’s symbiosis and its host-plant molecular interaction. Moreover, the comparative genomic investigation between *B. diazoefficiens* 113-2, *B. diazoefficiens* USDA110, *M. huakuii* 7653R, *Mesorhizobium japonicum* MAFF303099, *S. fredii* USDA205 and *S. meliloti* 2011 provided valuable insights into the species specificity and host specificity among different rhizobia.

## Results

### Complete Sequencing of the *B. diazoefficiens* 113-2 Genome

**FIGURE 1:**
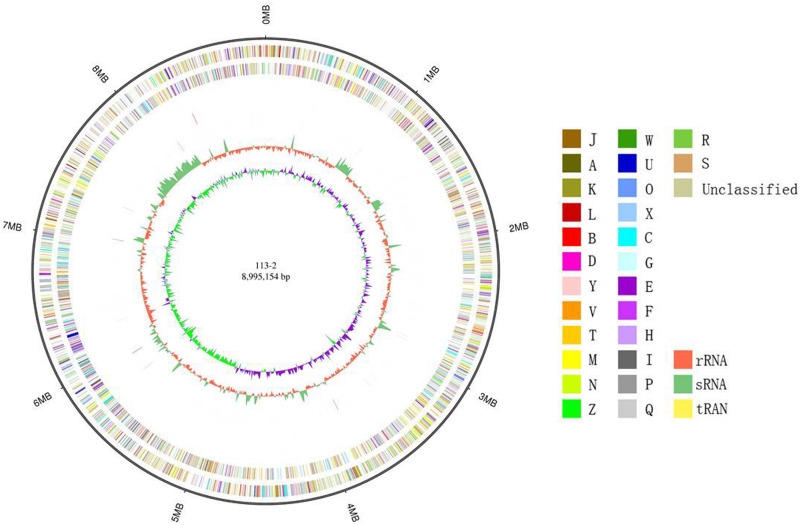
Circle map of the complete *B. diazoefficiens* 113-2 genome. Displayed circles from outer to inner: genome size (ring 1), forward strand gene, colored according to COG classification (ring 2), reverse strand gene, colored according to COG classification (ring 3), forward strand ncRNA (ring 4), reverse strand ncRNA (ring 5), repeat (ring 6), GC (ring 7), and GC-SKEW (ring 8).

In the present study, a PacBio RS II platform and Illumina HiSeq 4000 platform were used to sequence the genome of *B. diazoefficiens* 113-2 in order to systematically investigate this strain’s symbiosis and its host-plant molecular interactions. The total sequence of the *B. diazoefficiens* 113-2 genome was 8,995,154 bp in length, consisting of only one chromosome (Figure 1). The GC content of the whole genome was 64.1% and shown on the circle map of the *B. diazoefficiens* 113-2 genome (Figure 1). Table 1 summarizes previously sequenced main genome characteristics of *B. diazoefficiens* 113-2 as well as the genomes of five other strains (*B. diazoefficiens* USDA110, *M. huakuii* 7653R, *M. japonicum* MAFF303099, *S. fredii* USDA205 and *S. meliloti* 2011). These six strains belonged to different genera and had different host plants. *M. huakuii* 7653R, *M. japonicum* MAFF303099 and *S. meliloti* 2011 have two plasmids each, *B. diazoefficiens* 113-2 and *B. diazoefficiens* USDA110 have none, and *S. fredii* USDA205 only have 255 contigs.

**TABLE 1:** General feature of *B. diazoefficiens* 113-2 and five other rhizobia genomes.

	*B. diazoefficiens 113-2*	*B. diazoefficiens USDA110*	*M. huakuii 7653R*	*M. japonicum MAFF303099*	*S. fredii USDA205*	*S. meliloti 2011*
Growth rate	Slow	Slow	Medium slow	Medium slow	Fast	Fast
Host plant	Soybean	Soybean	*A.sinicus*	*Lotus*	Soybean	*Medicago* and *Trigonella*
Number of chromosomes/plasmids/contigs in genome	1	1	3	3	255 contigs	3
Genomic size(bp)	8995154	9105828	6881675	7596297	7152020	6693185
Genomic (G + C)%	64.1	64.1	63.3	62.51	62.2	62.16
Gene numbers in genome	8,801	8,502	6,301	7,107	6,909	6,315
tRNA in genome	50	53	51	52	51	55
rRNA in genome	3	3	4	6	15	9
Other RNA	41sRNA	4	4	4	4	4

In order to study the characteristics and functions of *B. diazoefficiens* 113-2, we analyzed most of its genomic components, including gene, non-coding RNA, repeat sequence and prophage (Table 2). We predicted 8,801 genes in the *B. diazoefficiens* 113-2 genome (Supplementary Table S1), which was the highest among the six genomes (Table 1). The numbers of genes were basically consistent with the trend of genome size. However, in the *Bradyrhizobium* genus, the genome size of *B. diazoefficiens* 113-2 was smaller compared with *B. diazoefficiens* USDA110, while its gene number was greater compared with *B. diazoefficiens* USDA110 (Table 1). We predicted the numbers and types of rRNAs, tRNAs, and sRNAs of *B. diazoefficiens* 113-2 genome (Table 2 and Supplementary Table S2), and found that both the soybean *Bradyrhizobium* genomes and *M. huakuii* 7653R and *M. japonicum* MAFF303099 genomes had essentially identical numbers of rRNAs and tRNAs, while *S. meliloti* 2011 and *S. fredii* USDA205 genomes had dramatically different numbers of these RNAs (Table 1). Besides, we examined the species composition in terms of tandem repeat sequences (Supplementary Table S3) and environmental adaptability-related prophage of *B. diazoefficiens* 113-2 genome (Table 2).

**TABLE 2:** Genome Component statistical analyses of *B. diazoefficiens* 113-2.

Genome components	Statistical analysis				
Gene	Total number	Total length (bp)	Average length	Length/genome length (%)	GC content
	8,801	7,635,828	867.61	84.89	64.88%
Non-coding RNA	Type	Copy number	Average length (bp)	Total length	In genome (%)
	tRNA	50	78	3,888	0.0432
	5s_rRNA (*De novo*)	1	114	114	0.0012
	16s_rRNA (*De novo*)	1	1,477	1,477	0.0164
	23s_rRNA (*De novo*)	1	2,872	2,872	0.0319
	sRNA	41	79	3,223	0.0358
Repeat sequence	Type	Number	Repeat size (bp)	Total length (bp)	In genome (%)
	TRF	389	3–828	41,105	0.457
	Minisatellite DNA	276	15–63	11,927	0.1326
	Microsatellite DNA	13	43,534	565	0.0063
Prophage	Phage length (bp)	Is complete?	Phage start (#)	Phage end (#)	GC content
1	13,798	Incomplete	4,990,661	5,004,458	65.47%
2	27,957	Incomplete	4,993,296	5,021,252	64.12%
3	7,794	Incomplete	7,773,295	7,781,088	59.25%

To evaluate the putative functions of *B. diazoefficiens* 113-2 gene set and provide clues for further research on finding target functional genes, we annotated the *B. diazoefficiens* 113-2 genome with 11 databases, including COG (Cluster of Orthologous Groups of proteins), GO (Gene Ontology), KEGG (Kyoto Encyclopedia of Genes and Genomes), NR (No-Redundant Protein Database), Swiss-Prot (O’Donovan et al., 2002), IPR, T3SS (Type III secretion system Effector protein), PHI (Pathogen Host Interactions), VFDB (virulence factor database), ARDB (Antibiotic Resistance Genes Database), and CAZY (Carbohydrate-Active enZYmes Database), and Supplementary Table S4 lists the detailed information. Supplementary Table S5 illustrates the number and proportion of different *B. diazoefficiens* 113-2 gene sets annotated in each database. The results showed that 97% genes of *B. diazoefficiens* 113-2 had annotated functions, and the length of the most unannotated genes was less than 500 bp, suggesting that almost all meaningful predictive genes of *B. diazoefficiens* 113-2 had annotated functions.

### Genome-Wide Synteny and ANI Analysis Among the Six Rhizobial Strains

**FIGURE 2:**
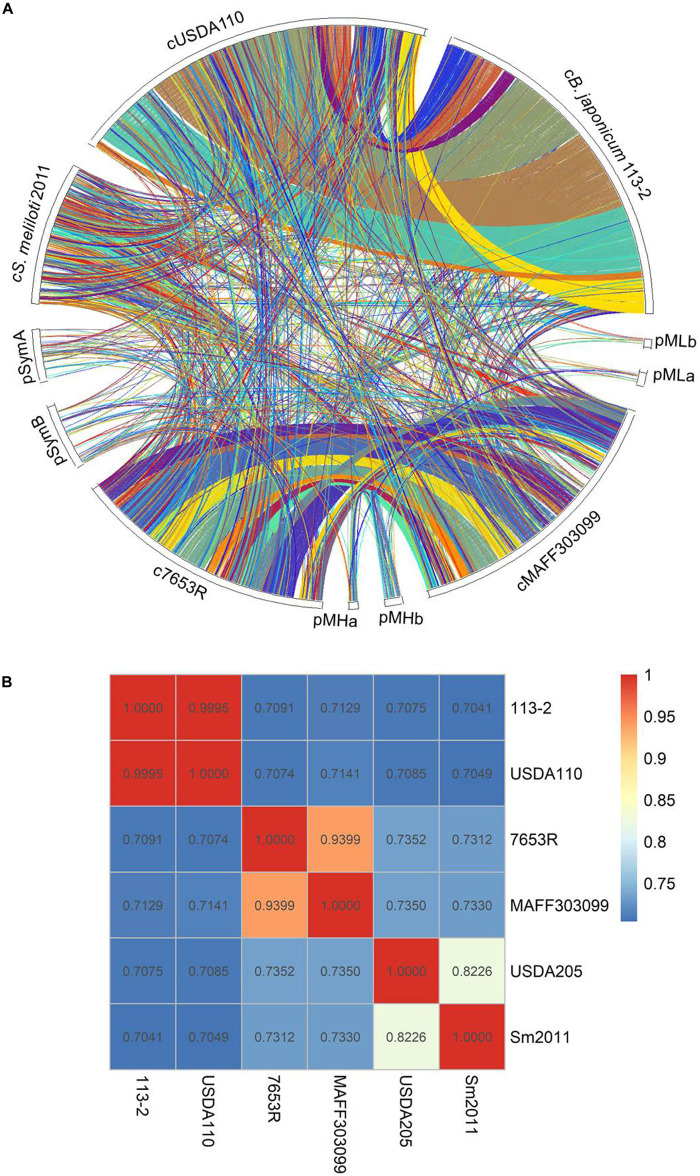
The circle map of genome synteny analysis and ANI analysis. **(A)** The circle map of genome synteny analysis among five rhizobial strains. Each colored block represents a synteny block and is internally independent from genomic rearrangement. **(B)** Summary of ANI calculations for the six rhizobial strains. The Average Nucleotide Identity (ANI) between the genome of *B. diazoefficiens* 113-2, *M. huakuii* 7653R, *M. japonicum* MAFF303099, *S. meliloti* 2011, *B. diazoefficiens* USDA110, and *S. fredii* USDA205 evaluated using the ANI Calculator.

To examine the phylogenetic relationships among the six strains, which belong to different genera and have different host plants, we firstly performed a synteny analysis based on the genome sequences of the above-mentioned five strains (except for *S. fredii* USDA205 with incompletely assembled genome) (Figure 2A). *B. diazoefficiens* 113-2 genome shared larger synteny blocks with *B. diazoefficiens* USDA110 compared with the other four strains. The gene consistency between the two strains in the same genus was higher than that in the different genera, and very few synteny blocks were shared between rhizobia of different genera.

Secondly, we carried out ANI analysis among these six rhizobial strains, and calculated the ANI values of each two rhizobial strains based on the nucleotide sequences (Figure 2B). *M. huakuii* 7653R and *M. japonicum* MAFF303099 were two strains in the same species (Wang et al., 2014), and the ANI value between these two strains was 0.9399. The ANI value between *B. diazoefficiens* 113-2 and *B. diazoefficiens* USDA110 was 0.9995, which even higher than that of *M. huakuii* 7653R and *M. japonicum* MAFF303099, indicating that these two strains were also in the same species. *S. fredii* USDA205 and *S. meliloti* 2011 were two strains in the fast-growing rhizobia genus, and the ANI value between these two strains was 0.8226. Besides, all of the ANI values between the two strains in different genera were less than 0.75, suggesting that relative lower correlation between the genomes of strains in different genera.

### Genome-Wide Ortholog Analysis Among the Six Rhizobial Strains

We compared the six genomes and identified the singletons of each strain and the numbers of shared clusters of each strain (Table 3). 1839 core-clusters were identified and similar numbers of shared variable-clusters predicted in the two strains in the same genus. There were significant differences in the proportions of singletons, which were mainly increased as the growth rate of bacteria was increased (Table 3). In the group of fast-growing rhizobia, about 19.3% (1,211) of the proteins in *S. meliloti* 2011 and 18.3% (1,094) in *S. fredii* USDA205 were singletons. In the medium-slow rhizobia group, about 13.9% (973) of the proteins in *M. japonicum* MAFF303099 and 10.3% (680) in *M. huakuii* 7653R were singletons. In the *Bradyrhizobium* genus, about 12.3% (1081) of the proteins in *B. diazoefficiens* 113-2 were singletons, while only 5.2% (416) in *B. diazoefficiens* USDA110 (Table 3), suggesting that *B. diazoefficiens* 113-2 had more unique functions compared with *B. diazoefficiens* USDA110. The detailed protein ID information of these singletons was shown in Supplementary Table S6.

**TABLE 3:** The cluster-singleton analysis of the six rhizobial strains genomes.

Species	Proteins	Co-clusters	Variable-clusters	Singletons
*B. diazoefficiens* 113-2	8801	1839	5661	1081
*B. diazoefficiens* USDA110	8070	1839	5662	416
*M. huakuii* 7653R	6571	1839	3728	680
*M. japonicum* MAFF303099	7018	1839	3951	973
*S. fredii* USDA205	5980	1839	2777	1094
*S. meliloti* 2011	6263	1839	2842	1211

**FIGURE 3:**
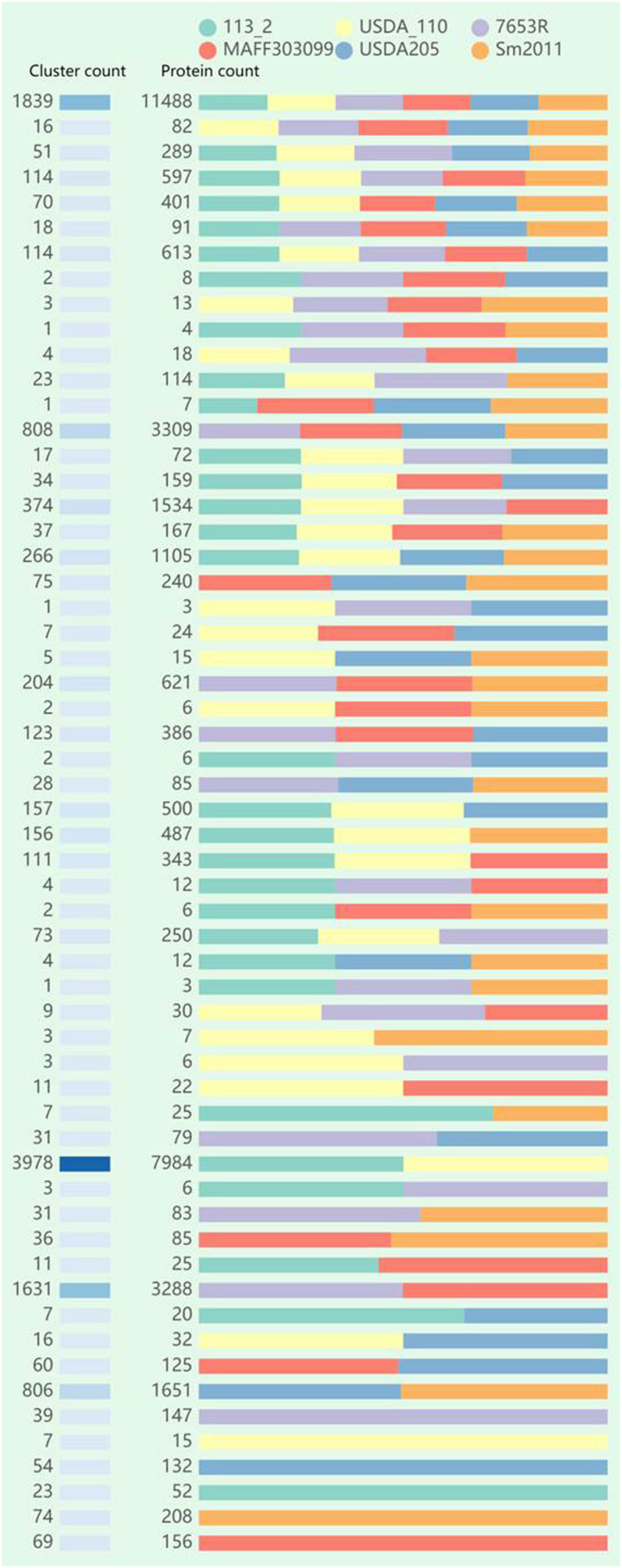
Summary of the distribution of orthologous clusters and protein data among the six rhizobial strains using OrthoVenn2.

We identified 11,656 clusters among the six rhizobia genomes (Figure 3), and the proteins in each cluster were shown in Supplementary Table S7. Of these clusters, 1839 (15.8%) clusters (including 1,615 single-copy gene clusters) were found to be shared by all of the six strains genomes and 266 clusters were existed in only one strain genome. An additional 383, 1,570, and 964 clusters were shared by five, four, and three of the six genomes, respectively. The remaining 6,634 clusters were observed to be present in two of the six genomes, among all these pair-wise comparisons, the 113-2-USDA110 pair was found to share the most abundant clusters (3978, 60.0%), followed by the 7653R-MAFF303099 (1,631, 24.6%) and *S. meliloti* 2011-*S. fredii* USDA205 (806, 12.1%), and there were very few clusters shared by the two genomes of strains in different genera.

### COG Function Classification of Singletons and Clusters Genes in the Six Rhizobial Strains

**FIGURE 4:**
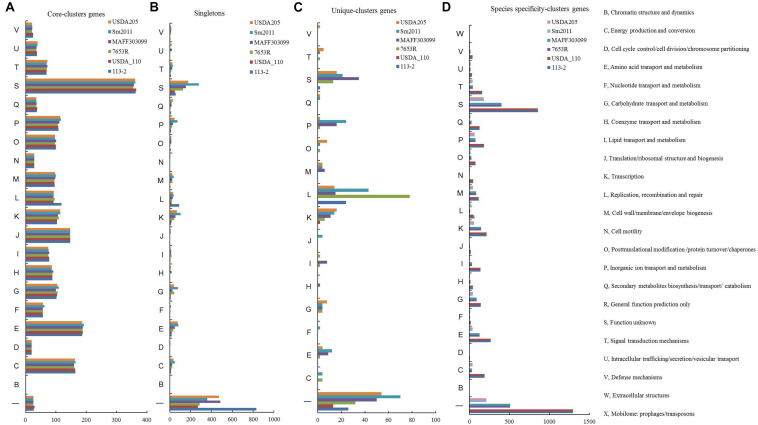
Cluster of Orthologous Groups of proteins (COG) functional classification of singletons and clusters genes in the six rhizobial strains. **(A)** COG functional classification of the core-clusters genes in the six rhizobial strains. **(B)** COG functional classification of singletons in the six rhizobial strains. **(C)** COG functional classification of the Unique-clusters genes in the six rhizobial strains. **(D)** COG functional classification of the species specificity-clusters genes in the six rhizobial strains.

To investigate whether the strain specificity and species specificity were related to the difference of protein numbers involved in various biological processes in rhizobia, we analyzed the COG assignments of the core-clusters genes, strain unique-clusters genes, species specificity-clusters genes and singletons (Figure 4), and Supplementary Table S8 lists the detailed gene ID information and annotation information. Similar numbers of core-clusters genes predicted in these COG functional terms were present in these six genomes, and the functions mainly focused on amino acid transport and metabolism (E), energy production and conversion (C), translation, ribosomal structure and biogenesis (J), Inorganic ion transport and metabolism (P), and Transcription (K) (Figure 4A), whereas there were significant differences in the numbers of singletons and strain unique-clusters genes (Figures 4B,C). The *B. diazoefficiens* 113-2 was found to have the highest proportion (about 82.1%) of the not annotated or Function unknown (S) singletons, and in most COG terms (15 out of 20), the numbers of singletons in *S. meliloti* 2011 or *S. fredii* USDA205 were more compared with the other strains, especially for energy production and conversion (C), amino acid transport and metabolism (E), carbohydrate transport and metabolism (G), transcription (K), and inorganic ion transport and metabolism (P) (Figure 4B). Compared with singletons, the strain unique-clusters genes did not assigned in the three COG functional terms (D, cell cycle control/cell division/chromosome partitioning; N, Cell motility; U, Intracellular trafficking/secretion/vesicular transport). All of the unique-clusters genes in *B. diazoefficiens* USDA110 were not annotated or Function unknown (S), half of the unique-clusters genes (26 out 52) in *B. diazoefficiens* 113-2 were annotated and predicted to have the function of Replication, recombination and repair (L), and more unique-clusters genes and more assigned COG functional terms of the rest four strains (Figure 4C). Similar gene numbers in these COG functional terms were present in the two strains of the same genus, while the numbers were decreased with the increase of growth rate (except for D, cell cycle control/cell division/chromosome partitioning; F, Nucleotide transport and metabolism; L, Replication, recombination and repair; N, Cell motility and U, intracellular trafficking/secretion/vesicular transport) (Figure 4D).

### KEGG Pathways Analysis of *B. diazoefficiens* 113-2

**FIGURE 5:**
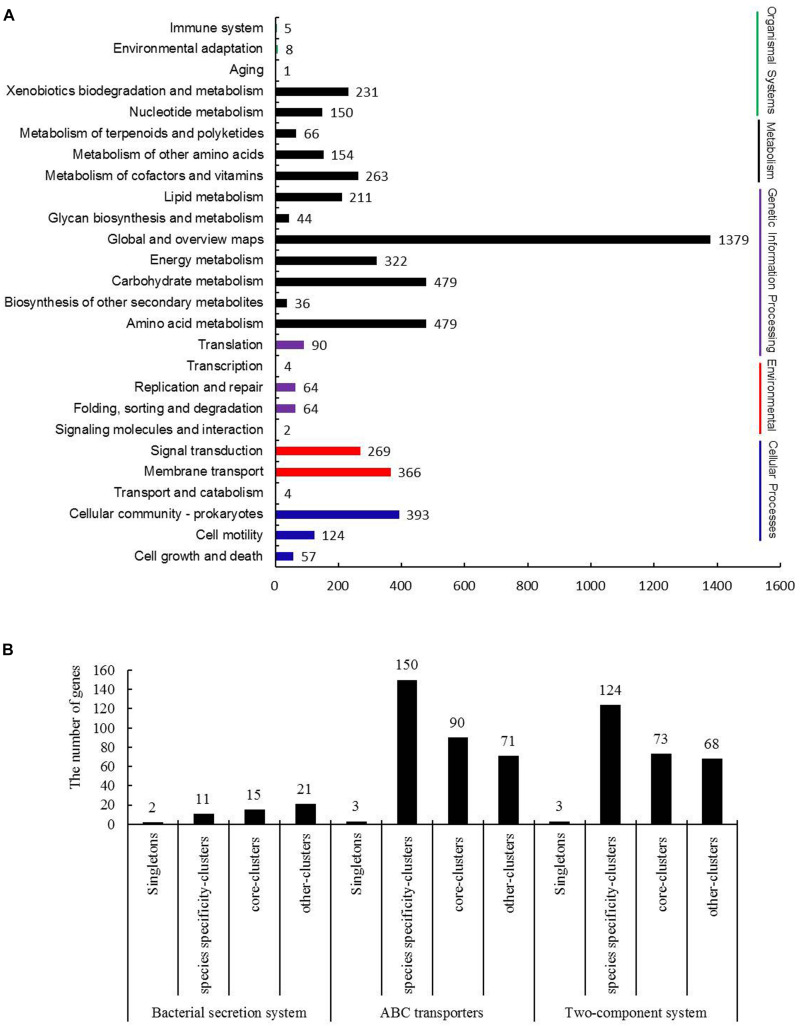
Kyoto Encyclopedia of Genes and Genomes (KEGG) pathway analysis of *B. diazoefficiens* 113-2. **(A)** KEGG annotation of *B. diazoefficiens* 113-2. **(B)** Three KEGG pathways analysis of singletons and clusters genes of *B. diazoefficiens* 113-2.

Kyoto Encyclopedia of Genes and Genomes is the major public pathway-related database, and a total of 26 KEGG pathways were listed in Figure 5A and divided into five categories as follows: cellular processes, environmental information processing, genetic information processing, metabolism and organismal systems. Most of the annotated genes were attributed to metabolism pathways, and the associated pathways primarily contained amino acid metabolism, carbohydrate metabolism, energy metabolism and global and overview maps. These results confirmed a preference for metabolism of amino acid, carbohydrates and energy.

To investigate whether the strain specificity and species specificity were related to the difference of protein numbers involved in various KEGG pathways in rhizobia, we mainly analyzed Bacterial secretion system (k03060), ABC transporters (k02010), and Two-Component system (k02020) of the core-clusters genes, strain unique-clusters genes, species specificity-clusters genes and singletons of *B. diazoefficiens* 113-2 (Figure 5B). The detailed gene ID information of the pathway genes was shown in Supplementary Table S9. The numbers of singletons for bacterial secretion system, ABC transporters and two-component system pathways were two, three and three, respectively, no strain unique-clusters genes for these three pathways, and about 27.3 ∼ 28.8% genes were core-clusters genes in these three pathways. The numbers of species specificity-clusters genes for bacterial secretion system was 11 (22.4%), however, the numbers for ABC transporters and two-component system pathways were 150 (47.8%) and 124 (46.3%), respectively (Figure 5B), indicating that the rhizobial genes in these two pathways were mainly species-specific.

### Host Specificity Analysis

The above-mentioned six genomes displayed drastically different host (Table 1). Because NFs, surface polysaccharides and secreted proteins are important determinants of host specificity of a rhizobium (Fauvart and Michiels, 2008), we explored genes that affect the biological synthesis of these signaling molecules in the genomes of these six strains.

#### Secretion System

**FIGURE 6:**
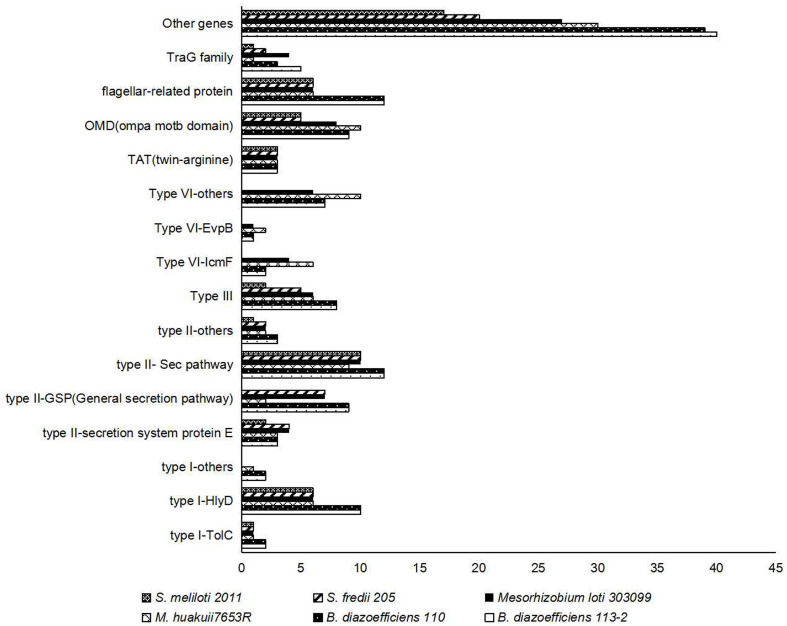
Numbers and distributions of genes related to different types of secretion systems in the six strain genomes.

Proteins secreted by rhizobial strains are necessary for beneficial symbiosis establishment (Wang et al., 2014). By means of gene families searches using secretion proteins of *B. diazoefficiens* 113-2 identified in the Bacterial secretion system (Figure 5B) as queries, we identified the genes related to secretory processes in the six strain genomes. We mainly analyzed two separate type-I systems, three type-II systems, type-III system, three type-VI systems, a twin-arginine (TAT) secretion system, a OmpA/MotB domain protein system, a flagellar-related protein system and a TraG family system (Figure 6). For the three strains that nodulate soybean, similar numbers of proteins in these analyzed secretion systems are present in *B. diazoefficiens* 113-2 and *B. diazoefficiens* USDA110, while *S. fredii* USDA205 had different types and numbers of secretion proteins with the other two strains. Different numbers of secreted proteins of most of the analyzed secretion systems (except for TolC, HlyD, TAT, and flagellar-related protein systems) were present in *M. huakuii* 7653R, *M. japonicum* MAFF303099, *S. fredii* USDA205, and *S. meliloti* 2011. Besides, *M. huakuii* 7653R had the same number of secreted proteins as *M. japonicum* MAFF303099 in type-III system, and *S. fredii* USDA205 had the same number of secreted proteins as *S. meliloti* 2011 in Sec pathway system. The detailed information of these secreted proteins was shown in Supplementary Table S10.

#### Surface Polysaccharides Biosynthesis

**FIGURE 7:**
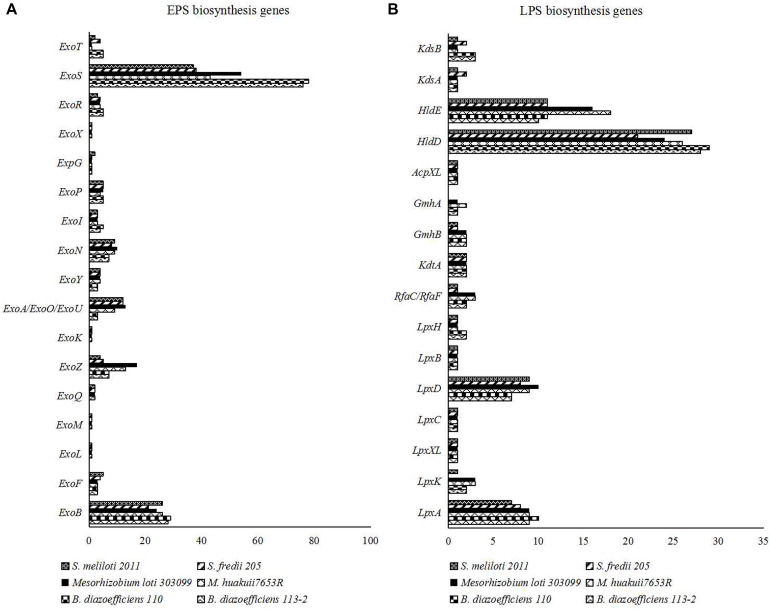
**(A)** Numbers of the genes related to the biosynthesis of EPSs in the six strain genomes. **(B)** Numbers of the genes related to the biosynthesis of LPSs in the six strain genomes.

Rhizobial cell-surface polysaccharidess, especially for exopolysaccharides (EPSs) and lipo- polysaccharides (LPSs), play important roles in establishing effective RNS with their hosts (Janczarek et al., 2010). We used the genes related to the biosynthesis of EPSs and LPSs identified in *M. huakuii* 7653R (Wang et al., 2014) as queries to identify the families of these genes. We explored and compared 19 EPS biosynthesis gene families (Figure 7A and Supplementary Table S11) and 17 LPS biosynthesis gene families in the genomes of the six strains (Figure 7B and Supplementary Table 11). Among them, four LPS biosynthesis gene (*LpxB*, *LpxC*, *LpxXL*, and *AcpXL*) families had the same numbers in all of the six strains. For the three strains that nodulate soybean, *S. fredii* USDA205 had different numbers of most of the genes related to surface polysaccharides biosynthesis with the other two strains. Besides, most of the genes had the similar numbers in the two strains of the same genus, while vary different between rhizobia of different genera. The detailed ID information of these genes related to surface polysaccharides biosynthesis was shown in Supplementary Table S11.

#### Nodulation, *nif* and *fix* Gene Family Analysis

Nodulation factors, which are produced by rhizobial strains in response to flavonoids secreted by legume root hairs, play key roles in the determinants of host specificity of a rhizobium. We applied the *nod*, *nif* and *fix* genes in *M. huakuii* 7653R and/or *M. japonicum* MAFF303099 (Wang et al., 2014) as queries to search the nodulation, *nif* and *fix* genes in the genomes of these six strains. We firstly identified and analyzed 39 NF families (22 *nod* gene families, 11 *nol* gene families, five *noe* gene families and one *nfe* gene family). Among them, nine gene families had core genes, and only *NodA* was a single-copy-core-ortholog among these genomes. Moreover, 25 gene families had unique genes, 26 gene families had species specificity genes, and 31 gene families had other types of genes (Table 4). Six genes (*NodF*, *NodH*, *NodQ*, *NolL*, *NolX*, and *NoeB*) did not exist in *B. diazoefficiens* 113-2 and *B. diazoefficiens* USDA110, two genes (*NodY* and *NoeB*) did not exist in *M. huakuii* 7653R and *M. japonicum* MAFF303099, and 11 genes (*NodB*, *NodY*, *NodZ*, *NolB*, *NolL*, *NolT*, *NolU*, *NolV*, *NolX*, *NolW*, and *NoeL*) were not found in *S. fredii* USDA205 and *S. meliloti 2011*. Besides, *NodY* did not exist in *B. diazoefficiens* 113-2, four genes (*NodU*, *NodZ*, *NolL*, and *NoeL*) were not found in *M. huakuii* 7653R, and two genes (*NodF* and *NoeB*) did not exist in *S. fredii* USDA205 (Table 4). Supplementary Table S12 lists the detailed gene information.

**TABLE 4:** List of the gene numbers of nodulation gene families among the six genomes.

Gene family	*B. diazoefficiens 113-2*	*B. diazoefficiens USDA110*	*M. huakuii 7653R*	*M. japonicum MAFF303099*	*S. fredii USDA205*	*S. meliloti 2011*
*NodA*	C(1)	C(1)	C(1)	C(1)	C(1)	C(1)
*NodB*	U(1)	U(1)	S(1)	S(1)		
*NodC*	O(1)	O(1)	O(1)	O(1)	U(1)	O(1)
*NodD*	S(3);O(1)	U(3);S(3);O(1)	U(2);O(3)	U(1);O(3)	U(4);S(1);O(1)	U(6);S(1);O(4)
*NodE*	C(1);U(1)	C(1);U(1)	C(1);O(1)	C(1);O(1)	C(1);O(1)	C(1);O(1)
*NodF*			O(2)	O(1)		O(1)
*NodG*	C(12);S(64);O(17)	C(12);U(1);S(65);O(20)	C(10);U(14);S(22);O(25)	C(10);U(15);S(22);O(27)	C(10);U(9);S(8);O(16)	C(12);U(25);S(8);O(22)
*NodH*			S(1);O(1)	S(1)	S(1)	U(1);S(1);O(1)
*NodI*	C(1);U(1);S(2);O(3)	C(1);U(1);S(2);O(4)	C(1);S(1);O(2)	C(1);S(1);O(2)	C(1);S(1);O(2)	C(1);S(1);O(3)
*NodJ*	C(1);S(4);O(1)	C(1);S(4);O(2)	C(1);S(1);O(1)	C(1);S(1);O(1)	C(1);S(2);O(2)	C(1);S(2);O(2)
*NodL*	S(1);O(1)	U(1);S(1);O(1)	S(3);O(3)	U(1);S(3);O(3)	S(1);O(3)	U(1);S(1);O(2)
*NodM*	O(2)	O(2)	U(1);S(2)	S(2);O(3)	S(1);O(1)	U(1);S(1);O(2)
*NodN*	C(1);U(1);S(8);O(1)	C(1);U(2);S(8);O(1)	C(1);U(1);S(2);O(4)	C(1);U(1);S(2);O(4)	C(1);S(1);O(1)	C(2);S(1);O(3)
*NodP*	S(2)	S(2)	S(1);O(1)	S(1);O(1)	S(1);O(2)	S(1);O(3)
*NodQ*			O(1)	O(1)	O(1)	O(2)
*NodS*	O(2)	U(1);O(3)	S(2);O(1)	U(2);S(2);O(1)	U(1);O(1)	U(3)
*NodT*	S(7);O(1)	U(2);S(7)	U(1);S(1)	S(1)	S(2);O(1)	S(3)
*NodU*	S(1);O(2)	S(1);O(2)		O(1)	U(2);S(1);O(2)	S(1)
*NodV*	S(11)	S(11)	S(4)	U(2);S(4)	S(2)	U(3);S(2)
*NodW*	S(8);O(4)	S(9);O(4)	U(2);O(1)	U(2);O(1)	U(3);S(1);O(2)	U(2);S(2);O(3)
*NodY*		U(1)				
*NodZ*	U(1)	O(1)		O(1)		
*NolA*	S(3)	S(3)	S(3);O(4)	S(3);O(4)	S(1);O(4)	U(2);S(2);O(4)
*NolB*	U(1)	U(1)	S(1)	S(1)		
*NolG*	S(10);O(10)	S(10);O(10)	U(1);S(3);O(6)	S(3);O(7)	U(1);S(2);O(7)	U(2);S(2);O(6)
*NolL*				U(3)		
*NolK*	C(3);S(16);O(5)	C(3);U(1);S(16);O(5)	C(3);U(4);S(9);O(6)	C(3);U(4);S(9);O(4)	C(3);U(2);S(9);O(2)	C(4);U(6);S(9);O(2)
*NolX*			S(1)	S(1)		
*NolR*	S(19);O(5)	U(1);S(19);O(5)	U(1);S(7);O(4)	U(4);S(7);O(9)	U(3);S(1);O(7)	U(3);S(1);O(8)
*NolT*	O(1)	O(1)	O(1)	O(1)		
*NolU*	O(1)	O(1)	O(1)	O(1)		
*NolV*	S(1);O(1)	S(1);O(1)	O(1)	O(1)		
*NolW*	O(1)	O(1)	O(1)	O(1)		
*NoeB*						U(1)
*NoeI*	C(1);O(1)	C(1);O(1)	C(1)	C(1)	C(1);O(1)	C(1)
*NoeK*	C(1);O(1)	C(1);O(2)	C(1);S(1)	C(1);S(2)	C(1);U(2)	C(1);O(1)
*NoeJ*	S(5);O(3)	U(1);S(5);O(3)	U(1);S(3)	U(4);S(3)	U(5);O(1)	U(2);O(1)
*NoeL*	O(2)	O(2)		O(1)		
*NfeD*	S(1);O(1)	S(1);O(1)	O(1)	O(2)	S(1);O(1)	S(1);O(1)

*C, core genes; U, unique genes; S, species specificity genes; O, other types genes.*

Secondly, the numbers of *nif* and *fix* genes were found to be different among these six genomes. Two *nif* gene families (*NifS* and *NifU*) and two *fix* gene families (*FixA* and *FixS*) had core genes, 13 gene families had unique genes, 14 gene families had species specificity genes, and 18 gene families had other types of genes (Table 5). *NifQ* had no ortholog in *S. fredii* USDA205 and *S. meliloti 2011*, *Nif11* was not found in *B. diazoefficiens* 113-2 and *S. meliloti 2011*, *NifW* had no ortholog in *M. huakuii* 7653R and *S. meliloti* 2011 genomes, and eight genes (*NifA*, *NifD/E/N/K*, *NifH*, *NifQ*, *NifT*, *NifX*, *NifZ*, and *FixU*) were not found in the *S. fredii* USDA205 genome. Two genes (*FixJ* and *FixK*) had larger numbers in *B. diazoefficiens* 113-2 and *B. diazoefficiens* USDA110 genomes compared with the other strains (Table 5). Table S13 lists the detailed gene information.

**TABLE 5:** List of the gene numbers of *nif*, *fix* gene families among the six genomes.

Gene family	*B. diazoefficiens* 113-2	*B. diazoefficiens* USDA110	*M. huakuii* 7653R	*M. japonicum* MAFF303099	*S. fredii* USDA205	*S. meliloti* 2011
*NifA*	O (1)	O (1)	U (2)	O (2)		U (1)
*NifB*	S (1); O (2)	S (1); O (2)	O (1)	O (1)	U (2)	O (2)
NifD/E/N/K	S (1); O (3)	S (1); O (3)	O (3)	O (4)		O (4)
*NifH*	O (1)	O (1)	O (1)	O (1)		O (1)
*NifQ*	S (1)	S (1)	U (1)	U (1)		
*NifS*	C (1)	C (1); O (1)	C (1)	C (1); O (1)	C (1); O (1)	C (1)
*NifT*	U (1); O (1)	U (1); O (1)	S (1); O (2)	S (1); O (1)		O (1)
*NifU*	C (1)	C (1)	C (1); U (1)	C (1)	C (1)	C (1)
*NifW*	O (1)	O (1)		O (1)	O (1)	
*NifX*	S (1)	S (1)	O (1)	O (1)		O (1)
*NifZ*	U (1); O (1)	U (1); O (1)	S (1); O (2)	S (1); O (1)		O (1)
*Nif11*		U (1)	U (1)	O (1)	O (1)	
*FixA*	C (1); O (1)	C (1); O (1)	C (1); O (1)	C (1); O (1)	C (2)	C (2); O (1)
*FixB*	O (2)	O (2)	O (2)	O (2)	U (2)	O (3)
*FixQ*	S (1)	S (1)	O (1)	O (2)	U (1); S (1)	S (1); O (2)
*FixJ*	S (8); O (4)	S (9); O (4)	U (2); O (1)	U (2); O (1)	U (3); S (1); O (2)	U (2); S (2); O (3)
*FixG*	S (2)	S (2)	O (1)	O (2)	O (1)	O (1)
*FixH*	S (1)	S (1)	S (1)	S (2)	S (1)	S (1)
*FixI*	S (1); O (1)	S (1); O (1)	O (2)	U (1); O (1)	U (1)	O (2)
*FixU*	U (1); O (1)	U (1); O (1)	S (1); O (2)	S (1); O (1)		O (1)
*FixK*	U (1); S (19); O (3)	U (2); S (19); O (3)	U (1); S (2); O (7)	U (4); S (2); O (5)	S (5); O (4)	U (6); S (4); O (5)
*FixS*	C (1)	C (1)	C (2)	C (2)	C (1); S (1)	C (1); S (1)

*C, core genes; U, unique genes; S, species specificity genes; O, other types genes.*

**FIGURE 8:**
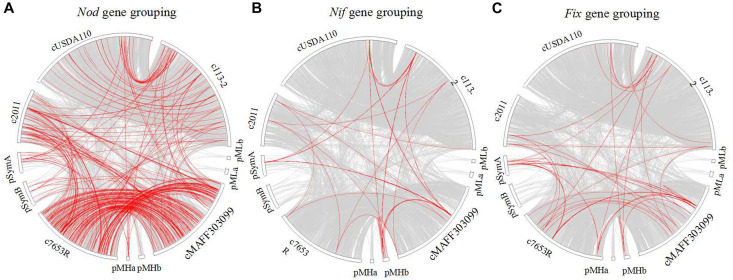
Synteny analysis of the *nod*, *nif*, and *fix* gene groupings among five rhizobial strains. Each gray block represents a synteny block and is internally independent from genomic rearrangement, red block represent *Nod* **(A)**, *Nif* **(B)**, and *Fix* **(C)** genes.

Thirdly, a synteny analysis based on the gene sequences of above-mentioned *nod* (Figure 8A), *nif* (Figure 8B), and *fix* (Figure 8C) genes (except for the genes in *S. fredii* USDA205) was performed to estimate the phylogenetic relationships of these genes among the five strains. In the three gene groupings, very few synteny blocks were shared by all of the five strains. The consistencies of the genes in the three groupings (especially for *nod* gene grouping) between the two strains in the same genus was higher than that in the different genera. Besides, we selected *NodW*, *NolK*, *NoeJ*, *NifB*, *FixK*, and *FixJ* gene families to perform phylogeny analyses (Supplementary Figures S1–S6), and the results revealed closer phylogenetic relationships between the two strains in the same genus, and only a small branch of *NodW* gene family especially for the three strains that nodulate soybean (Supplementary Figure S1).

## Discussion

The symbiotic nitrogen fixation system of leguminous plants and rhizobia is of great significance in the development of sustainable green agriculture. Although whole-genome sequencing of a series of rhizobial strains and comparative genomics among different rhizobial strains have provided valuable genetic information for symbiotic rhizobia (Tian et al., 2012; Sugawara et al., 2013; Wang et al., 2014), the genomic features responsible for species specificity among different rhizobial species with different growth rates still remain largely unexplored. Lots of genes (especially for *nod* genes) responsible for host specificity in the genomes of different strains have been explored by comparative genomics (Tian et al., 2012; Wang et al., 2014), while few studies on the homology classification analysis of the genes in these key gene families. In the present report, we sequenced and annotated the *B. diazoefficiens* 113-2 genome. The genomic characteristics of six rhizobia from different species and hosts were analyzed by comparative genomic analysis. Besides, the candidate genes related to secretion system, surface polysaccharides biosynthesis and RNS in the genomes of the six strains were explored and compared. Our results enriched the genomic library of rhizobia, and provided new insights and basic gene materials for species - specificity and strain - specificity of rhizobia.

### Genomic Evidence Supporting 113-2 as a Strain of *B. diazoefficiens*

*Bradyrhizobium diazoefficiens* 113-2 is a broad-host-range and highly efficient soybean rhizobium, and had higher symbiotic matching abilities than *B. diazoefficiens* USDA110 with soybean ‘Tianlong 1’ (Li et al., 2017a). The general feature and structure of *B. diazoefficiens* 113-2 genome were similar to *B. diazoefficiens* USDA110 (Table 1). *B. diazoefficiens* 113-2 genome shared a large proportion of synteny blocks and high ANI value (0.9995) with *B. diazoefficiens* USDA110 (Figure 2). Typically, the ANI values between genomes of the same species are above 95% (Goris et al., 2007). About 53% clusters in *B. diazoefficiens* 113-2 and *B. diazoefficiens* USDA110 genome were species specificity-clusters (113-2-USDA110 pair, Figure 3), and similar species specificity-clusters gene numbers in the assigned COG functional terms were present in these two genomes (Figure 4D). For nodulation, *nif* and *fix* genes, similar gene numbers of species specificity existed in *B. diazoefficiens* 113-2 and *B. diazoefficiens* USDA110 genomes (Tables 4, 5). These results supported a closer phylogenetic relationship between *B. diazoefficiens* 113-2 and *B. diazoefficiens* USDA110 compared with the other strains, and our current findings provided molecular evidence that *B. diazoefficiens* 113-2 and *B. diazoefficiens* USDA110 were two strains in the same species. Compared with *B. diazoefficiens 113-2*, *B. diazoefficiens* USDA110 contained more nodulation, *nif* and *fix* genes, including the unique genes in the nine nodulation gene families (*NodD*, *NodG*, *NodL*, *NodS*, *NodT*, *NodY*, *NolK*, *NolR*, and *NoeJ*) and *NifS* gene family (Tables 4, 5 and Supplementary Tables S12, S13), and these particular genes might be play key roles in the difference of the symbiotic matching abilities between *B. diazoefficiens* 113-2 and *B. diazoefficiens* USDA110 strains.

Compared with the other five genomes, *B. diazoefficiens* 113-2 had more small RNAs (Tables 1, 2 and Supplementary Table S2), which act as signal molecules modulating the host nodulation (Ren et al., 2019). Moreover, 1,081 (about 12.3%) singletons, which are unique genes of a species (Grose et al., 2014), were found in *B. diazoefficiens* 113-2 genome (Table 3), and most of them (831 out of 1,081) had no assigned COG functional terms (Figure 4B and Supplementary Table S8), suggesting that *B. diazoefficien*s 113-2 had more or unique functions compared with the other five strains. Besides, *B. diazoefficiens* 113-2 also had singletons in the selected KEGG pathways (Figure 5B) and had unique nodulation, *nif* and *fix* genes (Tables 4, 5), which are important for host specificity (Andrews and Andrews, 2017). These results suggested that *B. diazoefficiens* 113-2 had unique characteristics of genomic and symbiotic functions.

### Contrasting Genomic Features of Three Species of Rhizobia With Different Growth Rates

The development and maintenance process of legume-rhizobium symbiosis is a high resource-consuming process (Ferguson et al., 2019). Therefore, the equilibrium between the nitrogen fixation efficiency and energy consumption in legume-rhizobium symbiosis is particularly important in legume cultivation. To screen rhizobia with both high symbiotic efficiency and low energy consumption (fast growth rate and/or short cycle), we compared the genomic characteristics of three rhizobial species with different growth rates. Firstly, the genomic size, genomic (G + C)% and gene numbers were relatively consistent in the same genus, which were increased as the growth rate of bacteria was slowed down (Table 1). This finding was consistent with an earlier report (Tian et al., 2012). Secondly, similar genome structures and high ANI values were existed between the two strains in the same genus, while greatly varied genome structures and low ANI values were found among the strains in different genera with different growth rates (Figure 2). The difference in genomes might be the reason for the different symbiotic characters of different rhizobia (Siqueira et al., 2014) or rhizobia in different genera (Tian et al., 2012; Sugawara et al., 2013; Alaswad et al., 2019). Thirdly, the numbers of genus-specific cluster genes were decreased with the increase of growth rate of the strains in most of these COG functional terms (Figure 4C), indicating that there were more genes involved in various processes in slow-growing rhizobia. These genes would improve the ecological success of slow-growing rhizobia growing under more diverse soil conditions with limit but various resources (Konstantinidis and Tiedje, 2004; Tian et al., 2012), which might be the reason for that the adaptation of slow-growing rhizobia is wider compared with the other rhizobia (Tian et al., 2012). Fourthly, for candidate genes related to secretion system, surface polysaccharides biosynthesis and RNS, the numbers of genus-specific genes were relatively consistent in the strains of the same genus, while there were great differences among strains in different species of rhizobia (Figures 6–8 and Tables 4, 5). These differences might be the key factors to distinguish the host ranges as well as the nodulation and nitrogen fixation characteristics between rhizobia of different species (Tian et al., 2012; Wang et al., 2014; Zhang et al., 2014; Alaswad et al., 2019).

### Host Specificity

In most rhizobia, expression of genes related to secretion system, surface polysaccharides biosynthesis and RNS is needed for inducing nodule organogenesis and nodule development (Putnoky et al., 1988; Lorkiewicz, 1997; Fauvart and Michiels, 2008; Li et al., 2014), and the type and/or number of these nodule-related genes are often play important roles in host specificity (Horvath et al., 1986; Philip-Hollingsworth et al., 1989; Wang et al., 2014). Among our six tested strains, *S. fredii* USDA205 nodulated the same legume host (soybean) with *B. diazoefficiens* 113-2 and *B. diazoefficiens* USDA110 (Table 1), while there were no genes related to secretion system, surface polysaccharides biosynthesis and RNS that were both specific and common to these three strains (Figures 6, 7 and Tables 4, 5), suggesting that there was no gene specifically shared by rhizobia of different species to establish symbiosis with soybean, which was consistent with a previous study (Tian et al., 2012). *M. huakuii* 7653R and *S. meliloti* 2011 form indeterminate nodules (Cheng et al., 2007; Sallet et al., 2013), and the other four strains form determinate nodules (Kaneko et al., 2000; Yuan et al., 2016, 2017; Shah and Subramaniam, 2018). However, this phenomenon was consistent with the above-mentioned findings, and no gene was specifically shared by *M. huakuii* 7653R and *S. meliloti* 2011 or the rest four strains, indicating that the formation of determinate nodules or indeterminate nodules was mainly determined by host legume plants. The two strains nodulate different legume hosts in the group of medium-slow-growing rhizobia or fast-growing rhizobia (Table 1). In these four rhizobial strains, the types and total numbers of genes related to secretion system, surface polysaccharides biosynthesis and RNS were substantially different (Figures 6, 7 and Tables 4, 5). Among the RNS-related gene families, only 11 gene families (*Nod A*, *Nod E*, *Nod G*, *Nod I*, *Nod J*, *Nod P*, *Nod Q*, *Nol K*, *Nol R*, *Fix G*, and *Fix H*) had same gene types in these four strains, and three of them (*Nod G*, *Nol K*, and *Nol R*) had unique genes (Tables 4, 5). These differences might contribute to the establishment of differential legume-rhizobium symbiosis.

Collectively, the *B. diazoefficiens* 113-2 genome was sequenced, assembled and annotated in the present study. The synteny, ANI and ortholog analysis firmly establish 113-2 as a strain of *B. diazoefficiens*. The genomic characteristics of the six rhizobial strains from different species and different hosts were analyzed by comparative genomic analysis. The candidate genes related to secretion system, surface polysaccharides biosynthesis and RNS in the genomes of the six strains were explored and compared. Our results enriched the genomic library of rhizobia and provided valuable insights into the species-specificity and host specificity among different rhizobial strains.

## Materials and Methods

### Bacterial Strains and DNA Preparation

*Bradyrhizobium diazoefficiens* 113-2 (Stored in our lab) was cultured in YMA plate for 4 days at 28°C. Cells of *B. diazoefficiens* 113-2 were harvested by centrifugation at 1,3000 rpm for 30 min. Genomic DNA was extracted by Beijing Genomics Institute (BGI, Shenzhen, China) using a Genomic DNA Mini Preparation Kit.

### Genome Sequencing, Assembly and Component Prediction

*De novo* sequencing of *B. diazoefficiens* 113-2 genome was performed by BGI using PacBio RS II platform and Illumina HiSeq 4000 platform. The proportion of clean data (1,144 Mb) was 87.95% among the total acquired reads (1,301 Mb) in the Illumina platform. The proportion of Subreads Post Filter data (544,740,462 bp) was about 99.7% among the Polymerase Read Post Filter (546,403,010 bp) in the PacBio platform. The analysis results of 15-kmer (Supplementary Figure S7) and GC-depth (Supplementary Figure S8) indicated that the sequencing was of good quality. Sequence assembly was done with SOAP *de novo* (Luo et al., 2012). Glimmer 3.02^1^ with Hidden Markov models was used to perform the gene prediction of *B. diazoefficiens* 113-2 genome assembly. RNAmmer 1.2 (Lagesen et al., 2007), Rfam 9.1 (Gardner et al., 2009) and tRNA scan-SE (Lowe and Eddy, 1997) were used to identify tRNA, rRNA, and sRNAs in *B. diazoefficien*s 113-2 genome. The tandem repeats annotation was obtained using the Tandem Repeat Finder^2^. The prophages were predicted using the PHAST (PHAge Search Tool) (Grissa et al., 2007).

### Genome Annotation

Gene function annotation of *B. diazoefficiens* 113-2 was performed by using Basic Local Alignment Search Tool (BLAST) against 11 different databases. These databases are COG (Clusters of Orthologous Groups), GO (Gene Ontology), KEGG (Kyoto Encyclopedia of Genes and Genomes), NR (Non-Redundant Protein Database databases), Swiss-Prot (O’Donovan et al., 2002), IPR, Type III secretion system (T3SS), PHI (Pathogen Host Interactions), VFDB (Virulence Factors of Pathogenic Bacteria), ARDB (Antibiotic Resistance Genes Database), and CAZy (Carbohydrate-Active enZYmes Database).

### Synteny Analysis and ANI Analysis

The complete nucleotide sequences and genomic features of strains *B. diazoefficiens* USDA110, *M. huakuii* 7653R, *M. japonicum* MAFF303099, *S. fredii* USDA205 and *S. meliloti* 2011 were obtained from GenBank (accession numbers: USDA 110, NC_004463; 7653R, NC_002678, NC_002679, and NC_002682; MAFF 303099, NC_002678, NC_002679 and NC_002682; USDA205, GCA_009601405; *S. meliloti* 2011, NC_020528, NC_020527, and NC_020560). The sequences were organized according to their chromosomal origins of replication for intuitive comparison. Genome sequence alignments were created using NCBI BLAST + and visualized using MCScanX (Wang et al., 2012) and Mauve software. The Average Nucleotide Identity (ANI) between the six genomes was performed using the ANI Calculator, available at https://www.ezbiocloud.net/tools/ani (Yoon et al., 2017).

### Singletons-Clusters Analysis

Orthologous clustering analysis were performed with the web server OrthoVenn2 with bacteria group parameters and an *E*-value cutoff of 1e−5 (Xu et al., 2019). The protein FASTA file containing predicted protein sequences for strains *B. diazoefficiens* USDA110, *M. huakuii* 7653R, *M. japonicum* MAFF303099, *S. fredii* USDA205 and *S. meliloti* 2011 were used to predict the orthologous gene clusters.

### Core-Pan Genes Analysis

Core/Pan genes of above-mentioned six strains were clustered by the CD-HIT 4.66^3^ rapid clustering of similar proteins software (Edgar, 2004) with a threshold of 50% pairwise identity and 0.7 length difference cutoff in amino acid, and the final gene pool after clustered analysis is called the pan gene pool. Proteins existed in all of the six genomes in the clustering results act as the core gene pool. Proteins only existed in one genome are classified as the specific gene pool. The rest of the Pan proteins after removing core proteins are called the Dispensable gene pool.

### Phylogenetic Analysis

The different *NolKs* or *FixKs* were applied for multi-species phylogenetic analysis. Multiple alignments of the full-length deduced amino acid sequences of these genes were conducted with Clustal W program. And the multi-species phylogenetic tree was performed using MEGAX software (Kumar et al., 2018) with Neighbor-Joining (NJ) method, and bootstrap analysis was conduct educing 1,000 replicates with the p-distance model.

### Nucleotide Sequence Accession Numbers

Complete genome sequences of *B. diazoefficiens* 113-2 have been submitted to GenBank under the assigned accession number (CP055233).

## Data Availability Statement

The datasets generated for this study can be found in online repositories. The names of the repository/repositories and accession number(s) can be found in the article/Supplementary Material.

## Author Contributions

SY and XZ designed this work. SY wrote the manuscript. SY, RL, and YF performed most of the experiments and analysis. HC, CZ, YH, LC, QH, and DC contributed substantially to the completion of this work. All authors contributed to the article and approved the submitted version.

## Conflict of Interest

The authors declare that the research was conducted in the absence of any commercial or financial relationships that could be construed as a potential conflict of interest.

## Footnotes

1 http://ccb.jhu.edu/software/glimmer/index.shtml

2 http://tandem.bu.edu/trf/trf.html

3 http://weizhongli-lab.Org/cd-hit/

## Supplementary Material

Supplementary Figure 1 Phylogenetic relationships among the *NodWs* from the six strains.

Supplementary Figure 2 Phylogenetic relationships among the *NoeJs* from the six strains.

Supplementary Figure 3 Phylogenetic relationships among the *NolKs* from the six strains.

Supplementary Figure 4 Phylogenetic relationships among the *NifBs* from the six strains.

Supplementary Figure 5 Phylogenetic relationships among the *FixJs* from the six strains.

Supplementary Figure 6 Phylogenetic relationships among the *FixKs* from the six strains.

Supplementary Figure 7 15-mer analysis on sample.

Supplementary Figure 8 GC content and depth correlative analysis.

Supplementary Table 1 The sequence information of genes in *B. diazoefficiens* 113-2 genome.

Supplementary Table 2 The detail information of different types of RNAs in *B. diazoefficiens* 113-2 genome.

Supplementary Table 3 The detail information of Tandem Repeats in *B. diazoefficiens* 113-2 genome.

Supplementary Table 4 The annotation information of the *B. diazoefficiens* 113-2 genome in 11 different databases.

Supplementary Table 5 Function annotation assignment from different databases.

Supplementary Table 6 The detail protein ID information of the singletons of the six rhizobial strains.

Supplementary Table 7 The detail protein ID information in the 11656 clusters.

Supplementary Table 8 The detail gene ID information in COG classification and annotation information of the genes in the six rhizobia.

Supplementary Table 9 The detailed gene ID information of the three pathway genes in *B. diazoefficiens* 113-2 genome.

Supplementary Table 10 The detailed information of the genes related to different secretion system in the six genomes.

Supplementary Table 11 The detail ID information of the genes related to the biosynthesis of EPSs and LPSs in the six genomes.

Supplementary Table 12 The detail information of the genes of nodulation gene families in the six genomes.

Supplementary Table 13 The detail information of the genes of *nif*, *fix* gene families in the six genomes.
