# Supplementary material for: Whole-Genome Sequencing of Bradyrhizobium diazoefficiens 113-2 and Comparative Genomic Analysis Provide Molecular Insights Into Species Specificity and Host Specificity
Source: Front Microbiol. 2020 Nov 16;11:576800. doi: 10.3389/fmicb.2020.576800 (PMC7709874; doi:10.3389/fmicb.2020.576800)
Supplement: Supplementary Figure 1 — Phylogenetic relationships among the NodWs from the six strains. [file Data_Sheet_1.PDF]

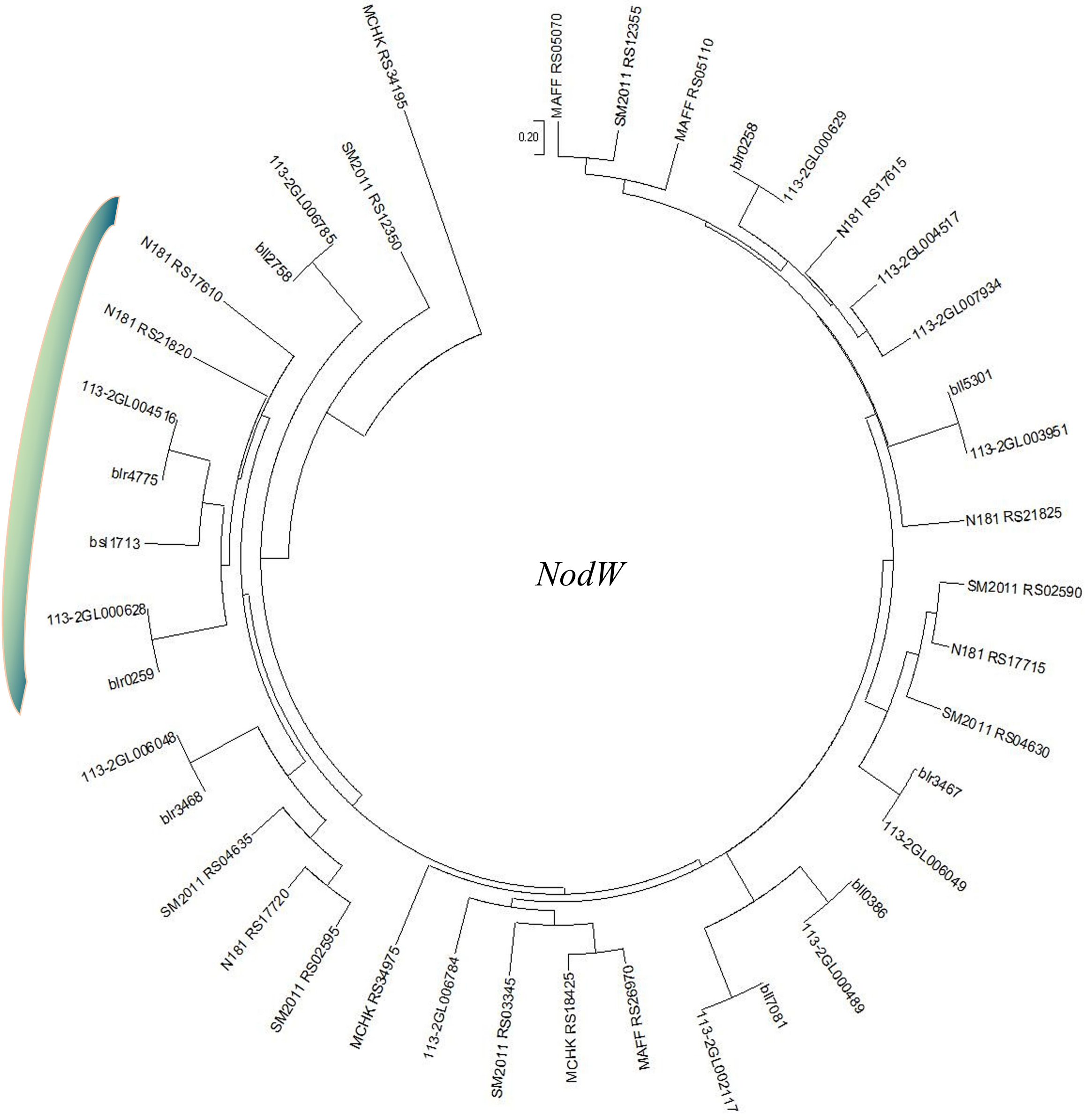

Figure S1: Phylogenetic relationships among the *NodWs* from the six strains.

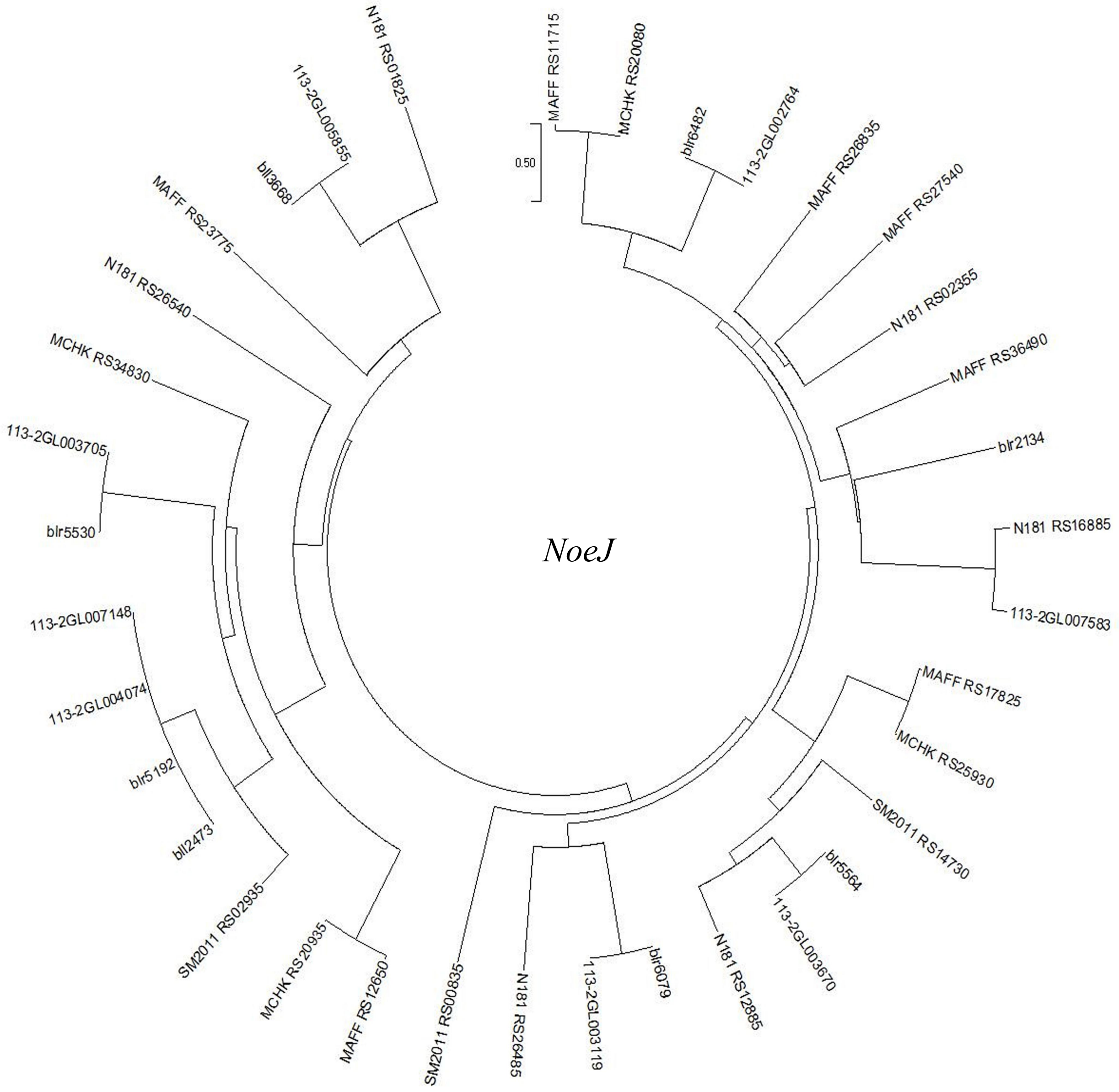

Figure S2: Phylogenetic relationships among the *NoeJs* from the six strains.

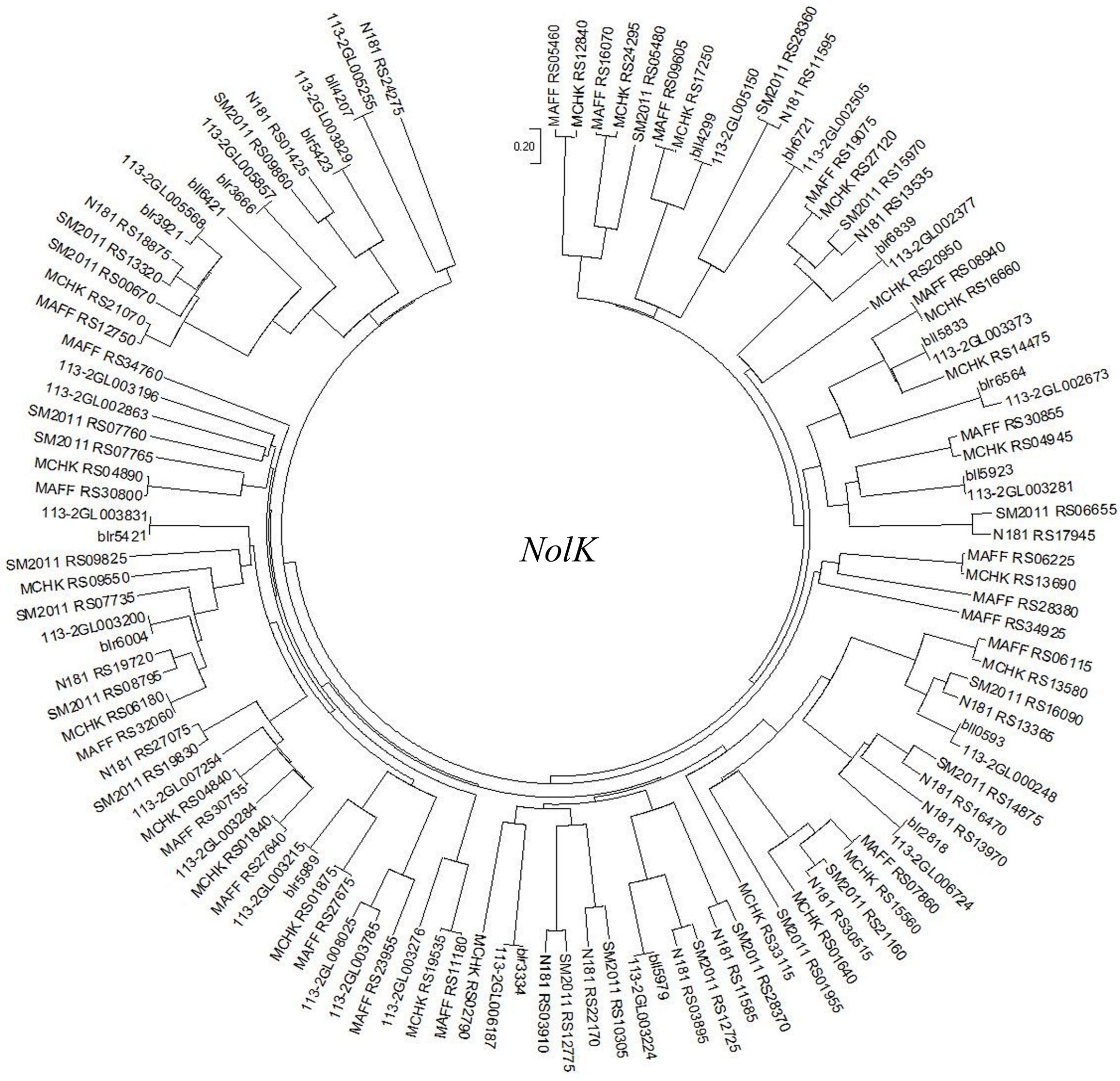

Figure S3: Phylogenetic relationships among the *NolKs* from the six strains.

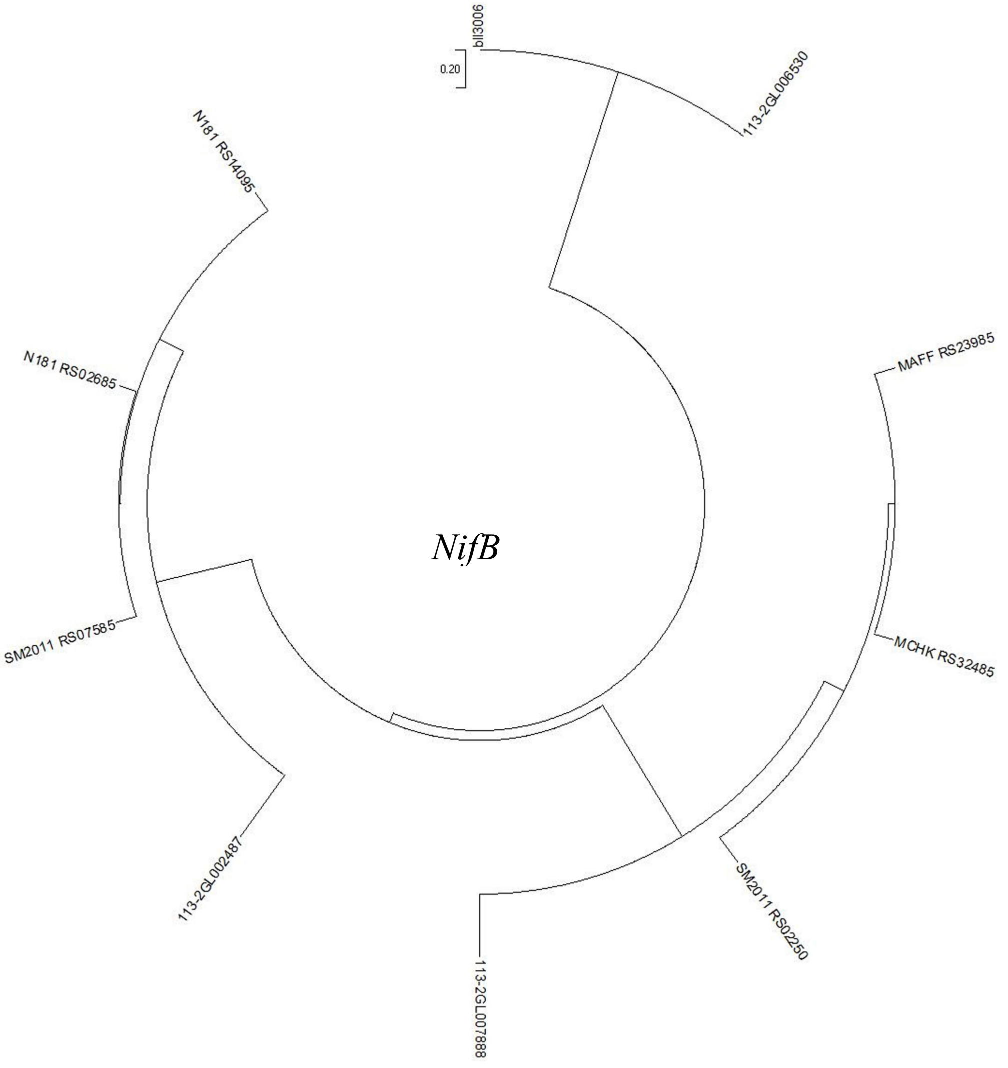

Figure S4: Phylogenetic relationships among the *NifBs* from the six strains.

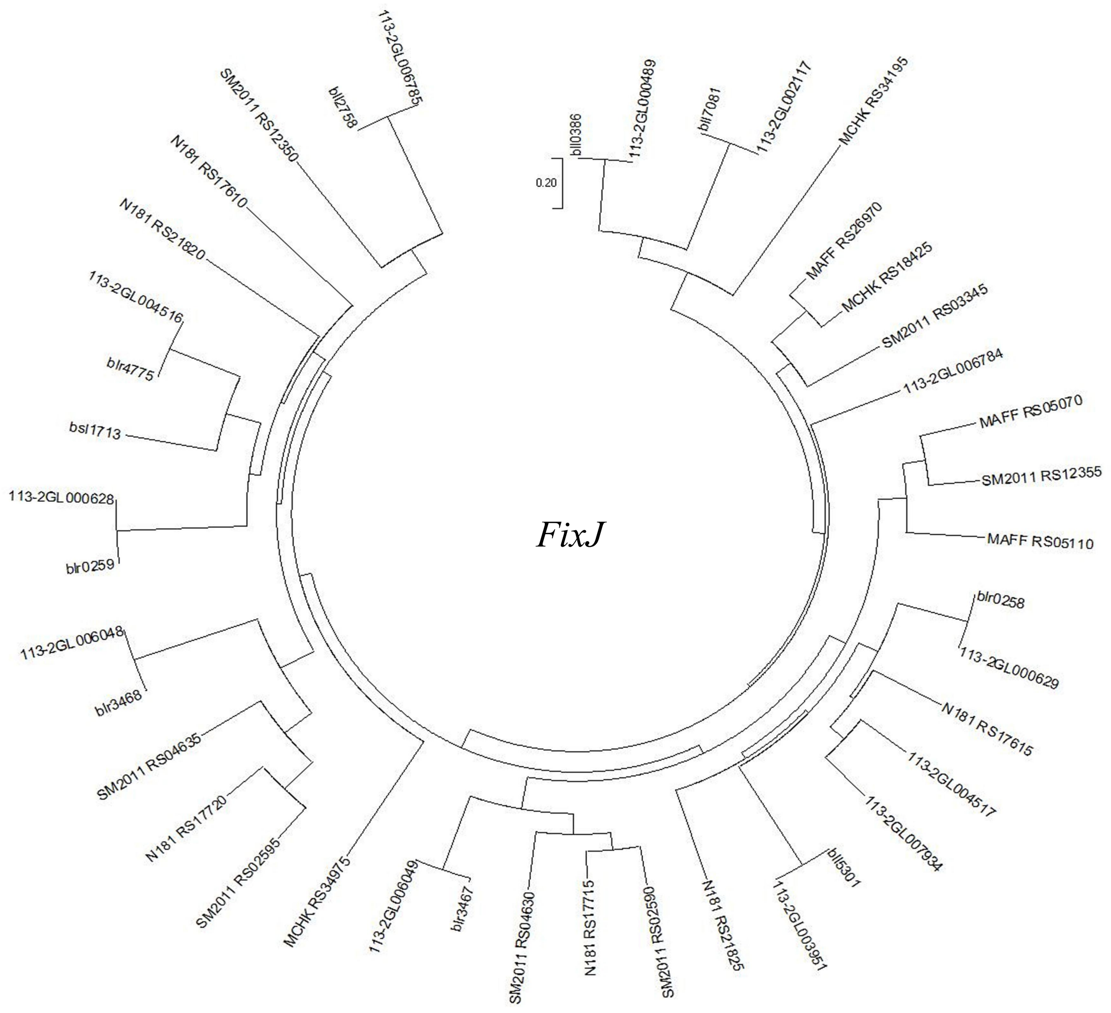

Figure S5: Phylogenetic relationships among the *FixJs* from the six strains.

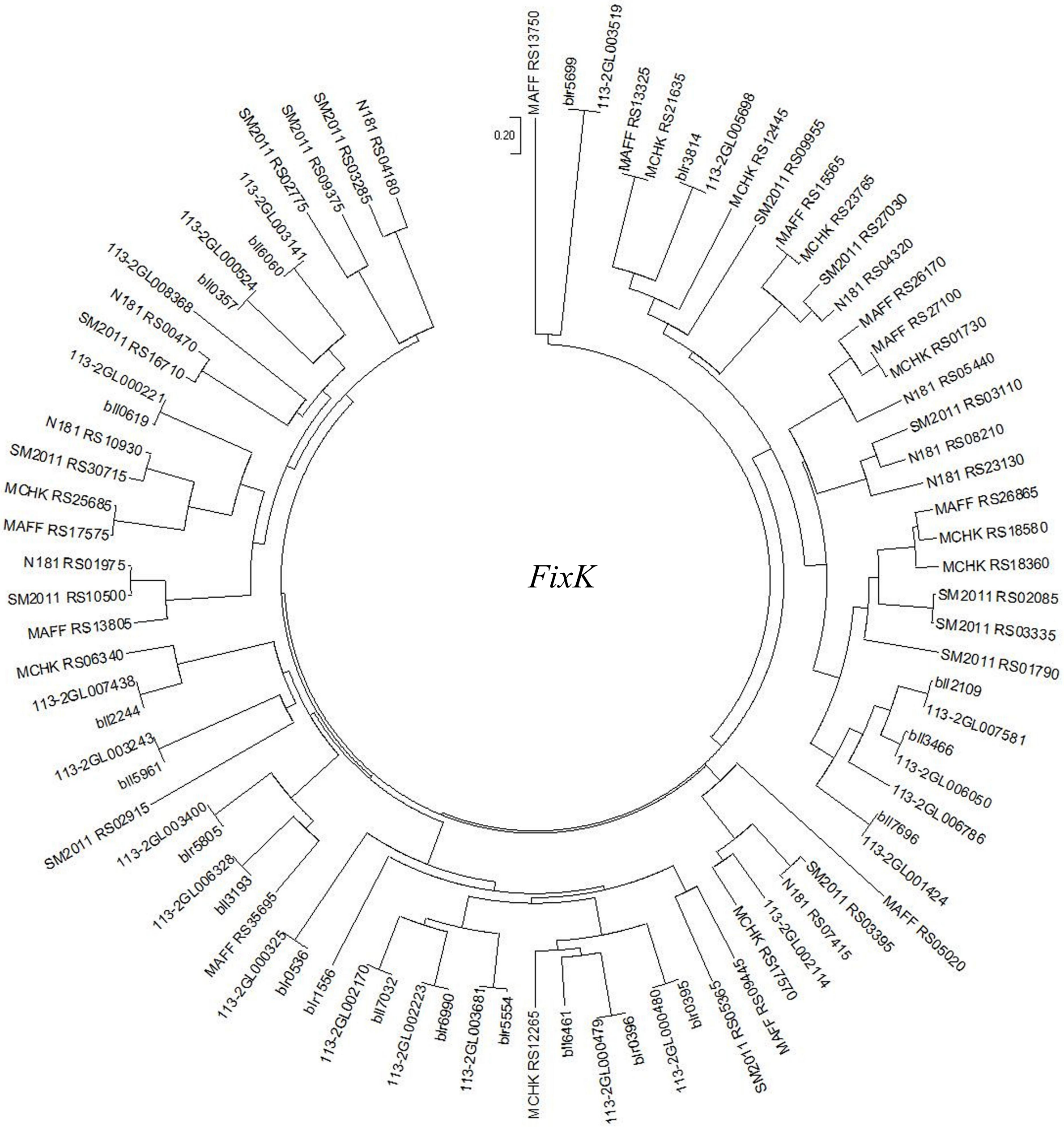

Figure S6: Phylogenetic relationships among the *FixKs* from the six strains.

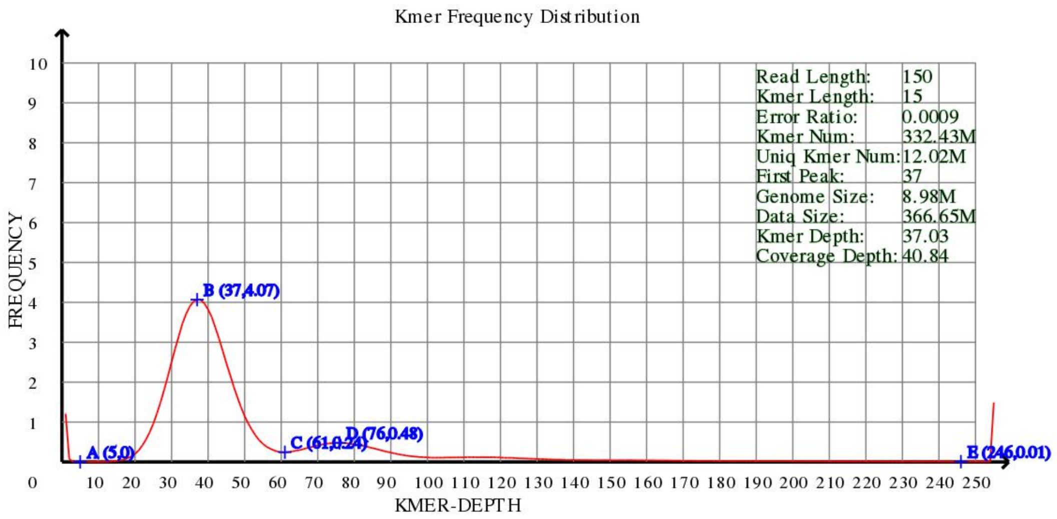

Figure S7: 15-mer analysis on sample. X-coordinate is depth, and Y-coordinate is proportion.

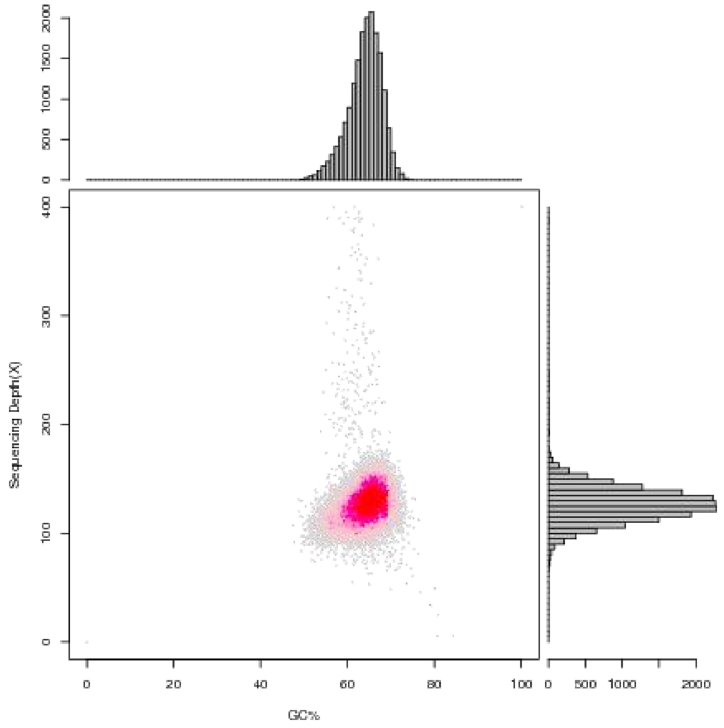

Figure S8: GC content and Depth correlative analysis. X-coordinate is GC content, and Y-coordinate is average depth.
